# Dating violence victimization across the teen years: Abuse frequency, number of abusive partners, and age at first occurrence

**DOI:** 10.1186/1471-2458-12-637

**Published:** 2012-08-10

**Authors:** Amy E Bonomi, Melissa L Anderson, Julianna Nemeth, Suzanne Bartle-Haring, Cynthia Buettner, Deborah Schipper

**Affiliations:** 1 Human Development and Family Science, The Ohio State University, Columbus, OH, USA; 2 Center for Injury Research and Policy, Nationwide Children’s Hospital, Columbus, OH, USA; 3 Group Health Research Institute, Seattle, WA, USA; 4 College of Public Health, The Ohio State University, Columbus, OH, USA; 5 Student Wellness Center, The Ohio State University, Columbus, OH, USA

**Keywords:** Abuse, Violence, Sexual abuse, Adolescence, Young adults

## Abstract

**Background:**

Prior longitudinal studies have shown high cumulative dating violence exposure rates among U.S adolescents, with 36 percent of males and 44 percent to 88 percent of females experiencing victimization across adolescence/young adulthood. Despite promising information characterizing adolescents’ dating violence experiences longitudinally, prior studies tended to concentrate on physical and sexual types of violence only, and did not report information on the number of times dating violence was experienced across multiple abusive partners. We used a method similar to the timeline follow-back interview to query adolescents about dating violence victimization from age 13 to 19—including dating violence types (physical, sexual, and psychological), frequency, age at first occurrence, and number of abusive partners.

**Methods:**

A total of 730 subjects were randomly sampled from university registrar records and invited to complete an online survey, which utilized methods similar to the timeline follow-back interview, to retrospectively assess relationship histories and dating violence victimization from age 13 to 19 (eight questions adapted from widely-used surveys covering physical, sexual, and psychological abuse). Then, for each dating violence type, we asked about the number of occurrences, number of abusive partners, and age at first occurrence. Of 341 subjects who completed the survey, we included 297 (64 percent females; 36 percent males) who had a dating partner from age 13 to 19.

**Results:**

Fully 64.7 percent of females and 61.7 percent of males reported dating violence victimization between age 13 and 19, with most experiencing multiple occurrences. More than one-third of abused females had two or more abusive partners: controlling behavior (35.6 percent); put downs/name calling (37.0); pressured sex (42.9); insults (44.3); slapped/hit (50.0); and threats (62.5). Males also had two or more abusive partners, as follows: controlling behavior (42.1 percent); insults (51.2); put downs (53.3); threats (55.6); and unwanted calls/texts/visits (60.7). Among abused females, 44.7 percent first experienced controlling behavior between age 13 and 15, whereas the majority (62.5 percent) first experienced pressured sex between age 16 and 17. Among males, for most abuse types, 16 percent to 30 percent of victimization began before age 15.

**Conclusions:**

Our study adds information to a substantial, but still growing, body of literature about dating violence frequency, age of occurrence, and number of abusive partners among adolescents.

## Background

Dating violence is widespread among adolescents in the United States, with cross-sectional studies showing that between 9% and 38% of adolescents have been victimized in the past year and/or within any dating relationship [1-13]. These studies have generally shown a gender symmetry trend for psychological and physical types of dating violence among adolescents. For example, Swahn’s study of adolescents recruited from a high risk, racially/ethnically diverse community showed that females and males, who reported on dating violence victimization within the last 12 months, experienced similar rates of psychological abuse (e.g., threats, insults, stalking) (38.3% among females versus 33.7% among males) and physical abuse (e.g., slapping, hitting, scratching, pushing, kicking, punching) (28.8% among females versus 32.6% among males) [7]. Further, studies showed that both females and males are sometimes victimized more than once by a dating partner; Coker’s findings showed, for example, that 3.2% of females and 1.2% of males had been beaten by a dating partner two to three times within the last 12 months [4]. Regarding sexual violence victimization, findings from cross-sectional studies have shown that sexual violence victimization rates tend to be higher among adolescent females (8.2% - 15.0%) compared to males (4.9% - 7.0%) [1,7] in recent dating relationships.

Studies have also captured adolescents’ longitudinal experience of *physical and/or sexual violence* beginning in adolescence through young adulthood [14-20]. Halpern and colleagues (2009) followed a longitudinal sample of males and females to determine physical and sexual violence victimization onset timing and persistence between adolescence and adulthood; their findings showed that 36% of males and 44% of females experienced victimization by adulthood and 7% of the total sample had persistent victimization from adolescence to adulthood [15]. Smith’s (2003) study, which included women age 18 and 19 recruited during their freshmen year in college, showed that girls victimized in high school were at significantly greater risk of revictimization in college, including risk of more than one type of victimization; overall, 88% of the sample experienced physical or sexual assault from age 14 through the fourth year of college and 63.5% experienced co-victimization [14]. Other longitudinal studies also showed similar trends of sexual and physical violence revictimization; once victimized in adolescence, subjects were at increased risk for revictimization in young adulthood/college years [16-20]. In addition to these studies, within the context of longitudinal intervention studies aimed at reducing dating violence, Foshee and colleagues showed that dating violence victimization could be reduced in males and females up to four years after the intervention was delivered [21]. In sum, these longitudinal studies were instrumental in adding to our understanding of how and when physical and sexual types of violence occur. However, the studies did not include psychological/emotional types of dating violence and similarly did not break down information about the number of dating violence occurrences and the number of abusive partners subjects had.

In addition to the high prevalence of dating violence among adolescents shown in U.S. studies and the tendency for re-victimization, as a public health concern, dating violence victimization has been shown to be associated with adverse mental and physical health problems, including depression, anxiety, suicide attempts, injuries, problem alcohol use and drug use, disordered eating, and risky sexual behavior [2-11,20]. Teens from racial and ethnic minority groups may be at disproportional risk for experiencing health burdens due to victimization. A study of 8,000 predominantly African American and Hispanic teens recruited from New York City high schools showed that dating violence victimization was among the top risk factors for females making a suicide attempt (61 percent more likely than non-victimized females) [5].

Despite the large body of extant literature documenting prevalence of dating violence victimization and health correlates, including longitudinal studies that have characterized adolescents’ experience of dating violence at multiple points in time [14-20], prior studies have not, to date, collectively characterized across the adolescent period (ages 13 to 19) all dating violence types (physical, sexual, and psychological/emotional), the number of times adolescents experience each of these abuse types, and the number of partners who perpetrated each abuse type. In the present investigation, we used a method similar to the timeline follow-back interview to query adolescents about their experiences of dating violence from age 13 to 19—including dating violence types, frequency, age at first occurrence, and number of abusive partners. The timeline follow-back assessment method has been widely used in studies to retrospectively capture at risk behaviors, such as drug and alcohol use, among adolescents [22-24]. We adapted the method to capture both relationship and dating violence histories in a sample of college students. While retrospective dating violence assessment may result in under-reporting of abuse due to issues of recall bias [25], retrospective assessment is the field’s standard for capturing dating violence experiences and our assessment method used memory prompts to facilitate recall, which we describe in more detail in the methods section.

The 2009 Institute of Medicine report – *Preventing Mental, Emotional, and Behavioral Disorders Among Young People* – called for a critical focus on the prevention of mental, emotional and behavioral disorders in young people [26]. Given that dating violence has been associated with mental and behavioral health issues that may be a result of the violence or a contributing factor to it, our study attempted to provide additional information about dating violence among adolescents spanning age 13 to 19, including the number of times they experienced the dating violence, the age they first experienced it, and number of abusive partners.

## Methods

Data for the study were collected as part of a feasibility study for testing the study questionnaire. The feasibility study was to serve as a platform for a larger longitudinal study that tracked dating violence experiences and health outcomes across subjects’ college/university career.

### Sample

Study procedures were approved by the institutional review board of The Ohio State University (Columbus, Ohio, United States). A random sample of 730 female and male students ages 18 to 21 enrolled at The Ohio State University on January 1, 2011 were identified through the registrar’s office to participate in a one-time only internet survey to assess dating violence experiences from age 13 to 19 and health. Subjects were credited $20 to their university student account for their participation in the study. During the first week of the academic spring quarter, using students’ university email account, we sent a recruitment email to all 730 students along with the study information sheet and link to the online survey. Two follow-up reminders were sent by email, three and seven days after the initial email, reminding students to complete the survey. The overall response rate at each recruitment email was as follows: initial email (31.6%, 231/730); second email (41.0%, 300/730); final email (46.7%, 341/730) — rates similar to prior studies of adolescents recruited using random sampling [27,28]. The response rate for the study was relatively low (46.7%), and we did not have information on non-respondents to assess potential response bias. However, our study respondents were remarkably similar to the Ohio State student population in general on critical socio-demographic factors, including age, race/ethnicity (17% comprised racial/ethnic minorities, consistent with the Ohio State population), and year in school (most comprised freshmen, sophomores and juniors).

Of the 341 subjects who completed the online questionnaire, 44 were excluded because they were older than age 21 (n = 7) or because they reported never having a dating, romantic or sexual partner between age 13 and 19 (n = 37). After these exclusions, the final analytic sample comprised 271 subjects (n = 190 females; n = 107 males).

### Survey

We used a method similar to the timeline follow-back interview to retrospectively assess subjects’ dating violence victimization histories from age 13 to 19 [22-24]; we had previously used this timeline follow-back interview method to document domestic violence and child abuse histories in more than 4,000 women and men [29-38]. As noted, while retrospective dating violence assessment may result in under-reporting of abuse due to issues of recall bias [25], retrospective assessment is the field’s standard for capturing dating violence experiences and our assessment method used memory prompts to facilitate recall. Namely, first, to establish relationship histories [29-38], subjects were asked whether they had a dating, romantic or sexual partner between age 13 and 19. They were then asked specific details about their three most recent partners, starting with their most recent partner, that is, the partner they were last involved with between age 13 and 19. For each of their three most recent partners, we asked about the gender of the partner, the age that the relationship began and ended, and the type of partnership (boyfriend/girlfriend; someone the subject liked romantically and went to specific events with, such as school dances or hung out with at the movies or mall; or someone the subject “hooked up with” or had a sexual relationship, but would not consider as a boyfriend or girlfriend) [39]. Similar to the timeline follow-back interview method, we used memory prompts, such as asking the subject to remember the year they were in high school to facilitate recall of the age that a relationship began and ended. For operational practicality, this relationship information was obtained for subjects’ three most recent partners, starting with their most recent partner and working back to include their two partners before that. Our decision to stop at asking detailed questions about subjects’ three most recent partnerships came from our extensive experience interviewing more than 4,000 adults, where subjects consistently expressed a desire to stop being asked beyond their third most recent relationship [29-38]. Of note, 63.4% (282 of 445) of females in our current adolescent sample and 70% (98 of 140) of males reported having three or fewer relationships from age 13 to 19. After we asked subjects these detailed questions about their three most recent partners, we asked about the total number of partners subjects had beyond the three most recent partnerships from age 13 to 19.

After information about subjects’ relationship history was gathered, dating violence victimization was assessed retrospectively using eight modified questions covering the three core conceptual areas of teen dating violence as recognized by the Centers for Disease Control and Prevention (physical, sexual, and psychological). Table 1 includes the questions as they were administered in our survey.

**Table 1:** Dating Violence Questions


**Has any partner you've been involved with between ages 13 and 19 ever…**
**Physical**
…hit, slap, or physically hurt you on purpose?
…threatened to hit or slap you, to spread rumors about you, to destroy something belonging to you, or to harm you in some other way?
**Sexual**
… pressured you to participate in sexual activities by begging or arguing with you, or by threatening to end your relationship?
… pressured you to participate in sexual activities by threatening you with physical force (i.e. twisting your arm or holding you down)?
**Psychological**
… tried to control your behavior by always checking up on you, telling you who your friends could be, or telling you what you could do and when?
… called you names, put down your looks, or said things to hurt your feelings on purpose?
… shouted, yelled, insulted, or sworn at you?
… made unwanted phone calls, send unwanted text messages, emails, or gifts, or showed up in person and waited for you when you didn't want them to?

Our eight questions were adapted from the Centers for Disease Control and Prevention’s Youth Risk Behavior Survey Surveillance System [40], Foshee and Swahn’s studies [1,7,13], and Coker’s teen dating violence survey currently being administered in a CDC-funded intervention study. As we were attempting to collect detailed information about subjects’ relationship histories, and dating violence frequency, number of abusive partners, and age at first occurrence spanning age 13 to 19, we used this collapsed set of eight questions, rather than a larger set of dating violence questions, such as those used in studies by Foshee and Swahn [1,7,13]. However, as noted, our collapsed questions covered the major conceptual areas of physical, sexual, and psychological types of dating violence typically measured in other questionnaires, and as reflected in the Centers for Disease Control’s conceptualization and the Teen Power and Control Wheel (a conceptual tool reflecting types of violence teens may experience in dating relationships). In a separate paper, we reported the results of a confirmatory factor analysis done on the eight dating violence questions included in our study, which predicted that the two sexual violence questions would load onto one factor, the four psychological abuse questions would load onto one factor, and finally that the two physical violence questions (including threats of physical violence) would load onto a single factor (Nemeth et al., under review). That analysis showed that all of the factor loadings were significant in the hypothesized direction, suggesting that the variables loaded onto the proper latent variables (sexual, psychological, and physical dating violence types).

For each question in Table 1, subjects were asked whether they ever experienced dating violence between age 13 and 19. For subjects who indicated they experienced any given dating violence type, they were then asked if 1) they experienced the violence in their last three relationships (reported on earlier in the survey) and any additional relationships beyond their most recent three; 2) the number of times they experienced each violence type; and 3) the age at first occurrence. For the two questions that addressed sexual violence, we asked about whether alcohol consumption was involved (partner, self, both, neither). For each dating violence type, subjects who responded with “yes” were considered exposed to that dating violence type.

### Data analysis

In gender-stratified analyses, we estimated overall dating violence prevalence (exposure was defined as a response of “yes” to any of the eight dating violence questions in Table 1), and then prevalence of each of the eight dating violence types (response of “yes” to each respective dating violence type signified exposure to that dating violence type). Confidence intervals and relative standard errors (RSE) were computed for the prevalence estimates. Prevalence estimates with RSE over 30% may be unreliable. Estimates with RSE > 30% are marked with an asterisk later in the results section and should be used with caution. Among subjects reporting dating violence, we then summarized the number of times the dating violence occurred, the number of partners the dating violence occurred with, and the age at first occurrence.

## Results

### Subject characteristics

Table 2 shows the characteristics of subjects. Consistent with The Ohio State University student population in general, the sample comprised mostly White subjects (83%). Most subjects reported heterosexual orientation (89.0% of females and 87.9% of males), and most were freshmen, sophomores or juniors from age 19 to 21.

**Table 2:** Subject Characteristics

	**Females (N = 190)**	**Males (N = 107)**
Age	N(%)	N(%)
18	28 (14.7)	15 (14.0)
19	58 (30.5)	39 (36.5)
20	62 (32.6)	25 (23.4)
21	42 (22.1)	28 (26.2)
Year in college		
Freshman	60 (31.9)	41 (38.3)
Sophomore	65 (34.6)	32 (29.9)
Junior	49 (26.1)	24 (22.4)
Senior	14 (7.5)	10 (9.4)
missing	2	0
GPA		
2.6 or lower	13 (6.8)	5 (4.7)
2.7 to 2.9	24 (12.6)	18 (17.0)
3.0 to 3.6	110 (57.9)	59 (55.7)
3.7 to 4.0	43 (22.6)	24 (22.6)
missing	0	1
Race		
White	156 (82.8)	89 (83.2)
Black	12 (6.4)	7 (6.5)
Asian	16 (8.5)	10 (9.4)
Other	5 (2.7)	1 (0.9)
missing	1	0
Sexual orientation		
Heterosexual	169 (89.0)	94 (87.9)
Bisexual	10 (5.3)	1 (0.9)
Homosexual	4 (2.1)	7 (6.5)
Asexual	7 (3.7)	5 (4.7)

† Missing responses not included in denominator when calculating percentages.

GPA=grade point average.

### Prevalence of dating violence

Table 3 depicts prevalence results. A total of 64.7% of females and 61.7% of males reported experiencing any type of dating violence from age 13 to 19.

**Table 3:** Prevalence of Dating Violence

**Female respondents (N = 190)**	**# reporting abuse**	**Prevalence**	**95% CI**	**RSE**
Any abuse	123	64.7	57.9, 71.6	5
Specific abuse types				
Controlling behavior	47	24.7	18.5, 30.9	13
Put downs, name calling	58	30.5	23.9, 37.1	11
Insults, yelling, swearing	81	42.6	35.5, 49.7	8
Unwanted calls, texts, visits	52	27.4	21.0, 33.8	12
Threats	17	9.0	4.9, 13.1	23
Hit, slap, physically hurt	7	3.7*	1.0, 6.4	37
Pressured into sex, begging	45	23.8	17.7, 29.9	13
Pressured into sex, threat of physical force	9	4.8*	1.7, 7.8	33
**Male respondents (N = 107)**	**# reporting abuse**	**Prevalence**	**95% CI**	**RSE**
Any abuse	66	61.7	52.3, 71.0	8
Specific abuse types				
Controlling behavior	20	18.7	11.2, 26.2	20
Put downs, name calling	16	15.0	8.1, 21.8	23
Insults, yelling, swearing	47	43.9	34.4, 53.5	11
Unwanted calls, texts, visits	29	27.1	18.5, 35.7	16
Threats	10	9.3*	3.7, 15.0	30
Hit, slap, physically hurt	14	13.1	6.6, 19.6	25
Pressured into sex, begging	12	11.2	5.1, 17.3	27
Pressured into sex, threat of physical force	1	1.0*	0.0, 2.8	100

*CI* = Confidence Interval; *RSE* = relative standard error.

*This estimate should be used with caution. Estimates with RSE > 30% may be unreliable.

Among females, psychological dating violence rates were: 24.7% (controlling behavior); 27.4% (unwanted calls/texts/visits); 30.5% (name calling/put downs); and 42.6% (insults/yelling/swearing). Nearly 25% of females experienced sexual pressure due to a partner’s persistent begging or threats and 5% due to physical force, 9% experienced threats of physical force, and 3.7% had been hit, slapped or otherwise physically hurt by a partner.

Among males, psychological dating violence rates were: 15.0% (put downs/name calling); 18.7% (controlling behavior); 27.1% (unwanted calls/texts/visits); and 43.9% (insults/yelling/swearing). A total of 11% of males experienced sexual pressure due to partner’s persistent begging or threats, and 1% due to physical force, 9.3% experienced threats of physical force and 13.1% had been hit, slapped or otherwise physically hurt by a partner.

### Number of abusive partners, number of dating violence occurrences, and Age at first occurrence

Tables 4 and 5 presents information on number of abusive partners, number of dating violence occurrences, and age at first occurrence.

**Table 4:** Dating Violence Chronicity (Female respondents)

**Female respondents (N=190)**	**Controlling behavior**	**Put downs, name calling**	**Yell, swore, insulted**	**Unwanted calls, texts, visits**	**Threaten**	**Hit, slapped, hurt**	**Pressured into sex, begging**	**Pressured into sex, threat**
Number reporting abuse	N=47	N=58	N=81	N=52	N=17	N=7	N=45	N=9
	n (%)†	n (%)†	n (%)†	n (%)†	n (%)†	n (%)†	n (%)†	n (%)†
Number of abusive partners								
1	29 (64.4)	34 (63.0)	44 (55.7)	32 (64.0)	6 (37.5)	3 (50.0)	24 (57.1)	5 (62.5)
2	13 (28.9)	15 (27.8)	25 (31.7)	12 (24.0)	6 (37.5)	3 (50.0)	11 (26.2)	2 (25.0)
3+	3 (6.7)	5 (9.3)	10 (12.7)	6 (12.0)	4 (25.0)	0 (0.0)	7 (16.7)	1 (12.5)
missing	2	4	2	2	1	1	3	1
Number of occurrences								
1	8 (17.0)	13 (23.2)	14 (17.3)	9 (18.0)	6 (35.3)	4 (66.7)	8 (18.2)	4 (50.0)
2-5	12 (25.5)	19 (33.9)	34 (42.0)	21 (42.0)	5 (29.4)	1 (16.7)	18 (40.9)	1 (12.5)
6-11	7 (14.9)	8 (14.3)	5 (6.2)	4 (8.0)	0 (0.0)	0 (0.0)	5 (11.4)	1 (12.5)
11-20	4 (8.5)	2 (3.6)	11 (13.6)	4 (8.0)	2 (11.8)	0 (0.0)	6 (13.6)	0 (0.0)
>20	6 (12.8)	4 (7.1)	7 (8.6)	8 (16.0)	3 (17.7)	1 (16.7)	5 (11.4)	2 (25.0)
don’t remember	10 (21.3)	10 (17.9)	10 (12.4)	4 (8.0)	1 (5.9)	0 (0.0)	2 (4.6)	0 (0.0)
missing	0	2	0	2	0	1	1	1
Age at first occurrence								
13-15	21 (44.7)	20 (35.1)	22 (27.2)	15 (28.9)	5 (29.4)	0 (0.0)	17 (38.6)	2 (25.0)
16-17	20 (42.6)	24 (42.1)	39 (48.2)	19 (36.5)	5 (29.4)	4 (66.7)	17 (38.6)	5 (62.5)
18-19	6 (12.8)	13 (22.8)	20 (24.7)	18 (34.6)	7 (41.2)	2 (33.3)	10 (22.7)	1 (12.5)
missing	0	1	0	0	0	1	1	1
Was alcohol involved								
No							31 (70.5)	5 (62.5)
Yes							13 (29.6)	3 (37.5)
missing							1	1

† Missing responses not included in denominator when calculating percentages.

**Table 5:** Dating Violence Chronicity (Male respondents)

**Male respondents (N=107)**	**Controlling behavior**	**Put downs, name calling**	**Yell, swore, insulted**	**Unwanted calls, texts, visits**	**Threaten**	**Hit, slapped, hurt**	**Pressured into sex, begging**	**Pressured into sex, threat**
Number reporting abuse	N=20	N=16	N=47	N=29	N=10	N=14	N=12	N=1
	n (%)†	n (%)†	n (%)†	n (%)†	n (%)†	n (%)†	n (%)†	n (%)†
Number of abusive partners								
1	11 (57.9)	7 (46.7)	21 (48.8)	11 (39.3)	4 (44.4)	8 (72.7)	6 (54.6)	1 (100.0)
2	5 (26.3)	7 (46.7)	19 (44.2)	15 (53.6)	5 (55.6)	2 (18.2)	4 (36.4)	0 (0.0)
3+	3 (15.8)	1 (6.7)	3 (7.0)	2 (7.1)	0 (0.0)	1 (9.1)	1 (9.1)	0 (0.0)
missing	1	1	4	1	1	3	1	0
Number of occurrences								
1	4 (20.0)	0 (0.0)	8 (17.4)	5 (17.9)	0 (0.0)	4 (33.3)	6 (50.0)	1 (100.0)
2-5	8 (40.0)	4 (26.7)	21 (45.7)	9 (32.1)	8 (88.9)	8 (66.7)	4 (33.3)	0 (0.0)
6-11	2 (10.0)	6 (40.0)	4 (8.7)	2 (7.1)	0 (0.0)	0 (0.0)	1 (8.3)	0 (0.0)
11-20	1 (5.0)	2 (13.3)	4 (8.7)	5 (17.9)	1 (11.1)	0 (0.0)	0 (0.0)	0 (0.0)
>20	3 (15.0)	2 (13.3)	4 (8.7)	4 (14.3)	0 (0.0)	0 (0.0)	0 (0.0)	0 (0.0)
don’t remember	2 (10.0)	1 (6.7)	5 (10.9)	3 (10.7)	0 (0.0)	0 (0.0)	1 (8.3)	0 (0.0)
missing	0	1	1	1	1	2	0	0
Age at first occurrence								
13-15	6 (30.0)	9 (60.0)	13 (28.9)	8 (28.6)	2 (22.2)	2 (18.2)	2 (16.7)	0 (0.0)
16-17	7 (35.0)	5 (33.3)	23 (51.1)	10 (35.7)	3 (33.3)	5 (45.5)	5 (41.7)	0 (0.0)
18-19	7 (35.0)	1 (6.7)	9 (20.0)	10 (35.7)	4 (44.4)	4 (36.4)	5 (41.7)	1 (100.0)
missing	0	1	2	1	1	3	0	0
Was alcohol involved								
No							8 (66.7)	0 (0.0)
Yes							4 (33.3)	1 (100.0)
missing							0	0

† Missing responses not included in denominator when calculating percentages.

#### Number of abusive partners

In contrast to our studies on adults where most women and men indicated they had only one abusive partner [29,38], teens tended to report that more than one partner perpetrated dating violence toward them. Among females who reported dating violence, more than one-third indicated that they had experienced the dating violence from two or more partners, as follows: controlling behavior (35.6%); put downs/name calling (37.0%); pressured sex (42.9%); insults (44.3%); slapped/hit (50.0%); and threats (62.5%). A sizable proportion of males who experienced dating violence also said they had two or more partners who perpetrated the violence, as follows: controlling behavior (42.1%); insults (51.2%); put downs (53.3%); threats (55.6%); and unwanted calls/text messages (60.7%).

#### Number of dating violence occurrences

Among female and males reporting violence, dating violence was rarely reported as an isolated incident. Dating violence was commonly experienced as 2 to 5 occurrences of each dating violence type. However, of note, roughly 15% of females and males reported 20 or more occurrences of some dating violence types. Namely, for females, 16.0% experienced 20 or more occurrences of unwanted calls/texts/visits, 17.7% experienced threats, 16.7% were hit, and 25.0% were pressured sexually using physical force. For males, 15.0% experienced 20 or more occurrences of controlling behavior and 14.3% experienced unwanted calls/texts/visits.

#### Age at first occurrence from age 13 to 19

Some dating violence types tended to occur at earlier ages than other dating violence types. For example, among females reporting dating violence, 44.7% reported that they first experienced controlling behavior between the ages of 13 and 15, whereas the majority of females (62.5%) reporting being pressured into sex due to threats or physical force first experienced this type of dating violence between the ages of 16-17. Among males, age 13 to 15 was the most common age at earliest occurrence of put downs/name calling (60.0%). For most other dating violence types, between 16% and 30% of victimization began before age 15.

## Discussion

Our study used an assessment approach similar to the timeline-follow back method [22-24] to estimate dating violence victimization across the teen years (age 13 to 19), including dating violence types (physical, sexual, and psychological/emotional), frequency, age at first occurrence, and number of abusive partners. Our retrospective query approach included memory prompts, such asking subjects to remember what year they were in high school when they began and ended a relationship, to facilitate recall of relationship start and stop times and dating violence exposure. Our dating violence questions were adapted from prior surveys conducted by the Centers for Disease Control and Prevention (Youth Risk Behavior Surveillance System) [40], Foshee and Swahn [1,7,13], and Coker’s teen dating violence survey currently being administered in a CDC-funded intervention study. Across age 13 to 19, in our sample, 64.7% of females and 61.7% of males experienced dating violence victimization; our prevalence rates fall within the range of prior longitudinal studies showing cumulative physical/sexual dating violence victimization exposure during adolescence/early adulthood of 36% for males and 44% to 88% for females [14,15]. Differences in the way dating violence was measured in our study (retrospective assessment of physical, sexual, and psychological/emotional violence) versus in prior longitudinal studies (assessment of physical and sexual violence) could account for prevalence rate differences.

Our study was not powered to statistically compare prevalence rates between males and females. This said, our study generally showed that females and males tended to experience comparable rates of threats (9%), unwanted calls/texts/visits (27%), and being yelled/sworn at (43%), but females experienced higher rates of being put down and called names (30.5% versus 15.0%) and sexual pressure due to persistent begging or threats (23.8% versus 11.2%). Our findings corroborate results from prior studies showing higher rates of sexual violence victimization in females compared to males and similar rates of psychological abuse among adolescent females and males in dating relationships [1,4,7,15]. In contrast to some prior studies that have shown a general gender symmetry trend for physical dating violence victimization among adolescents[7], our study showed a higher rate of physical violence among males (13.1%) compared to females (3.7%). While these gender differences were observed in our study, we caution readers about over-interpreting for two reasons: 1) we did not undertake a formal statistical comparison due to our small sample size; and 2) we did not have qualitative information about the nature of the physical violence—it is possible, for example, that in our predominantly heterosexual sample males experienced “open hand slapping” by their female partners, where females experienced more aggressive physical abuse by male partners. Lending support to this idea, prior studies have shown that males are more likely than females to report physically injuring a date 7.

Our findings on the age at first dating violence occurrence, number of occurrences, and number of abusive partners add to our understanding of how dating violence unfolds during the teen years. Females and males rarely reported an isolated incident of dating violence. While teens most commonly experienced 2 to 5 occurrences of dating violence, of note, roughly 15% of both females and males reported 20+ occurrences of some dating violence types (for females: unwanted calls/texts/visits, threats, being hit, pressured sexually using physical force; for males: controlling behavior, unwanted calls/texts/visits). The age at first occurrence tended to be similar for males and females, with a few exceptions. Females tended to report controlling behavior earlier than males, and males tended to report put down and name calling earlier than females. In general, the first occurrence of pressure to have sex, and threats and physical harm tended to occur later than first occurrences of psychological dating violence such as controlling behavior, or name calling.

Our study results must be considered within the context of its limitations. First, generalizability is compromised due to our sample of young adults—predominantly White (83%)—enrolled at a large Midwestern university. While the racial/ethnic breakdown of our sample mirrors that of the university, our sample is less diverse than that of the U.S. population generally [41]. Studies have shown higher rates of dating violence among African American compared to White adolescents [1,8]. Our dating violence prevalence results from a predominantly White sample may therefore be conservative. Second, males in our sample were under-represented. Third, with a response rate of 46.7%, it is possible that respondents differed from non-respondents in meaningful ways. Unfortunately, we did not have information on non-responders; therefore an assessment of response bias was not possible. It is not possible to determine whether those who responded to the survey were more or less likely to have a history of teen dating violence compared to non-responders. However, in our study of adults where we had limited data on non-respondents, we performed a propensity score analysis to estimate the probability that a woman responded to the survey, based on age, length of enrollment in the health plan, and health care utilization in the year prior to the survey [29]. This analysis showed that the estimated probability of survey participation did not differ for women exposed to intimate partner violence compared to women who reported no intimate partner violence (estimated probability 0.58 vs. 0.57 respectively). Fourth, although retrospective dating violence assessment is the field’s standard for assessing dating violence, it is possible that subjects did not accurately recall dating violence they experienced. We attempted to minimize recall bias by using a query method similar the timeline follow-back interview [22-24], which included starting with recent events and working back and which included memory prompts to facilitate subjects’ recall. Even with this detailed assessment approach, it is possible that subjects mis-estimated dating violence they experienced [25].

## Conclusions

We used a query approach similar to the timeline follow-back interview method to facilitate recall of dating violence victimization experiences in a sample of males and females. Our study documented dating violence victimization experiences across the teen years, from age 13 to 19, including dating violence types, frequency, number of abusive partners, and age at first occurrence—providing important information for health professionals and others to respond to a very common problem among teens, even those at higher socioeconomic levels who go on to college. Our results point to the need to amplify primary and secondary prevention efforts; school-based programs have been effective in reducing dating violence occurrence in adolescents [1,21,42-49]. As well, with females experiencing sexual pressure at high rates and by multiple partners, there must be a concerted effort to discuss sexual health, including healthy relationships, healthy sexual boundaries, and consent; health care settings offer a safe, confidential place for such conversations.

## Competing interests

The authors declare they have no competing financial or non-financial interests.

## Authors’ contributions

AEB conceptualized the study and survey, oversaw data collection and analysis, and wrote the manuscript with co-authors’ input. MA helped design the survey, conducted the data analysis and critically reviewed the manuscript. JMN helped conceptualize the study and survey, added to the statistical analysis, and critically reviewed the manuscript. SBH, CB, and DS helped conceptualize the study and survey, and critically reviewed the manuscript. All authors read and approved the final manuscript.

## Pre-publication history

The pre-publication history for this paper can be accessed here:

http://www.biomedcentral.com/1471-2458/12/637/prepub
